# Hedging Our Bets: The Expected Contribution of Species to Future Phylogenetic Diversity

**Published:** 2007-09-25

**Authors:** Mike Steel, Aki Mimoto, Arne Ø. Mooers

**Affiliations:** 1 Biomathematics Research Centre, University of Canterbury, Christchurch, New Zealand; 2 IRMACS, Simon Fraser University, Burnaby, Canada; 3 Institute for Advanced Study, Berlin, Germany

**Keywords:** phylogenetic diversity, extinction, biodiversity conservation, Shapley index

## Abstract

If predictions for species extinctions hold, then the ‘tree of life’ today may be quite different to that in (say) 100 years. We describe a technique to quantify how much each species is likely to contribute to future biodiversity, as measured by its expected contribution to phylogenetic diversity. Our approach considers all possible scenarios for the set of species that will be extant at some future time, and weights them according to their likelihood under an independent (but not identical) distribution on species extinctions. Although the number of extinction scenarios can typically be very large, we show that there is a simple algorithm that will quickly compute this index. The method is implemented and applied to the prosimian primates as a test case, and the associated species ranking is compared to a related measure (the ‘Shapley index’). We describe indices for rooted and unrooted trees, and a modification that also includes the focal taxon’s probability of extinction and which links two complementary approaches to conserving phylogenetic diversity.

## Introduction

Within a given taxonomic group, individual biological species are generally considered to be of equal or near-equal biodiversity value. So, for instance, areas with a greater number of species are more valuable than those with fewer (Myers et al. 2000). When wild species are ranked by value, this is usually based on their threat of extinction (see e.g. SARA, 2002). However, as pointed out by Cousins (1991), species are discovered and identified because they are different from other species, which suggests that they may differ in value. In the context of conservation, Avise (2005) has highlighted five different currencies for valuing species: rarity, distribution, ecology, charisma, and phylogeny. Here, we consider the value of a species based on its position in a phylogeny. A phylogeny is the directional, acyclic graph depicting relationships between leaves (species), which we define formally in the next section. A phylogeny generally has a root (which assigns direction) and edge weights that can represent unique feature diversity (e.g. as measured by evolutionary time or genetic distance). Species can be defined by the features they possess, and one measure of their worth is the expected contribution of their genetic, morphological or evolutionary distinctiveness. To this end, we can use a phylogeny to assign a measure of evolutionary value to a species, since branch lengths can be chosen to correspond to genetic, morphological or evolutionary distance. Because of the highly imbalanced shape of the Tree of Life, some species in a phylogeny will have far fewer close relatives than others in that phylogeny (Mooers and Heard, 1997), and these more distantly-related species will be expected to contribute more unique features (Faith, 1992).

Phylogenetic measures of conservation value have a long pedigree (see e.g. Altschul and Lipman, 1990; May, 1990) and have begun to be explored in some detail (Haake et al. 2005; Hartmann and Steel, 2007; Pavoine et al. 2005a, 2005b; Redding and Mooers, 2006). So, for example, Pavoine and colleagues presented one new phylogenetic measure of conservation value, a set of sampling weights such that the expected pairwise distance on the tree is maximized. Haake and colleagues extended the ‘Shapley value’ (Shapley, 1953) from co-operative game theory to the conservation setting to calculate the average distance of a focal species to all possible subsets of taxa. For both measures, species with high conservation scores are those expected to contribute more to the resulting sets. Yet another measure that uniquely apportions the tree to its tips (Redding, 2003; Isaac et al. 2007) and which is the focus of a new international conservation initiative (the EDGE initiative, Zoological Society of London) scales almost perfectly with the Shapley value (unpublished results).

One question with these measures concerns the set of species that individual species are asked to complement. For instance, given known extinction probabilities for species, some future sets of species are much more likely than others and so some species will be more valuable because their close relatives are less likely to be included in future sets of species. Here we formalize this idea to extend the Shapley value of a species to include preassigned extinction probabilities, and show how this value can be efficiently computed. We then compare our measure with the original Shapley value using the prosimian primates as a test case.

In this paper we consider only extinction (neglecting possible speciation), because our focus is on the impact of current high rates of extinction over relatively short time frames (hundreds of years) for which little speciation may be expected. Also, although our indices rank species for conservation, we emphasise that such conservation includes safeguarding natural habitats.

**Figure 1.**
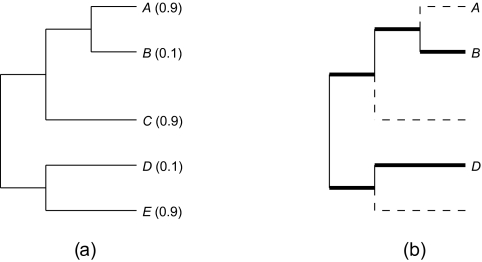
(**a**) A small rooted tree with edge lengths (of 2 units for the terminal edges incident with C, D, E, and 1 unit for the other five edges). Each tip *j* has an associated extinction probability *P*(*ext*) = **ɛ***_j_* . (**b**) For a subset *S* = {*B*, *D*} of taxa that are extant at some future time, the phylogenetic diversity score *PD*(*S*) is the sum of the lengths of the edges indicated in bold. The dashed edges lead to extinct taxa.

**Definition** Let T be a rooted or unrooted phylogenetic tree with leaf set *X*, together with an assignment of positive lengths to the edges (branches) of T. We let *l*(*e*) denote the length of edge *e*, and let *E*( T) denote the set of edges of T. For a subset *S* of *X*, let *PD* (*S*) denote the *phylogenetic diversity* of *S* defined as follows. If T is unrooted then *PD*(*S*) is the sum of the lengths of the edges (branches) of T in the minimal subtree that connects *S*. If T is rooted, then *PD*(*S*) is the sum of the lengths of the edges of T in the minimal subtree of T that connects *S* and the root of the tree. Figure 1 illustrates these concepts, and includes values at the tips that we will use in the next section. Note that although the branch lengths in this example are clock-like, this assumption is not required in any of the results we describe.

## The HED Index

For a leaf *i* ∈*X*, and a subset *S* ⊆ *X* – {*i*} let

$$\Delta_{PD}(S,\;i)\;:\;=\;PD\left(S\;\cup \;\{i\}\right)\;-\;PD\left(S\right).$$

The quantity Δ*_PD_* (*S*, *i*) measures how much phylogenetic diversity *i* contributes to the tree that one obtains from T once species not in *S* have been pruned out (for example if they go extinct). Alternatively, Δ*_PD_* (*S*, *i*) is the marginal increase in phylogenetic diversity of *S* if *i* is added.

Now, suppose that each species has an associated extinction probability *P*(*ext*) (which may vary from species to species)—for example, this may be the probability that the species is extinct in (say) 100 years from now (either globally, or in some specified community). We will denote this *P*(*ext*) value for species *j* by **ɛ***_j_*. In this paper we consider the simplest model which assumes that the extinction of each species in *X* comprise independent events. Given *i* ∈ *X*, let ${\text{S}}_{i}$ denote the random subset of species in *X* – {*i*} which survive (i.e. do not go extinct).

By the independence assumption we have:

$$P[{\text{S}}_{i}\;=\;S]\;=\;\prod_{j\in S}\left(1\;-\;{\text{ɛ}}_{j}\right)\times \;\prod_{j\in X-\{i\}-S}{\text{ɛ}}_{j}.$$

For *i* ∈ *X*, let *ψ_i_* denote the expected value of Δ*_PD_*( ${\text{S}}_{i}$, *i*). That is,

(1) $$\begin{array}{l} \psi_{i}\;=\;E\;[\Delta_{PD}\;({\text{S}}_{i},\;i)]\\ \;\;\;\;\;\;\;\;=\;\sum_{S\subseteq X\;-\;\{i\}}P\;[{\text{S}}_{i}\;=\;S]\;\Delta_{PD}\;(S,\;i). \end{array}$$

We call *ψ_i_* the *heightened evolutionary distinctiveness* of species *i*, and the function *i* ↦ *ψ_i_* the *heightened evolutionary distinctiveness* (HED) index for T. Notice that if all the species in *X* – {*i*} were guaranteed to survive, then *ψ_i_* would be just the length of the pendant edge incident with leaf *i*, however random extinctions mean that *ψ_i_* will tend to be increased (‘heightened’) over this pendant edge length.

The quantity *ψ_i_* measures the expected additional phylogenetic diversity species *i* would contribute at some future time if it is extant rather than extinct, given our uncertainty about which other species may have also be extinct at this future time. It is an example of a type of ‘distinctiveness’ measure described by Equation (2) of Weitzmann (1998). Notice that the extinction risk of species *i* does not influence *ψ_i_*, as this quantity depends only on the extinction risks of other species. We will discuss this issue (and describe a related index that does incorporate the extinction probability of species *i*) later in the paper.

A related but different index, based on the Shapley value in co-operative game theory, has recently been described by Haake et al. (2005). This index, denoted here as *ψ*^sh^ can be defined (for unrooted trees) as follows: For *i* ∈ *X*,

(2) $$\psi_{i}^{\text{sh}}\;=\;\frac{1}{\left|X\right|}\;\sum_{S\subseteq X-\{i\}}{\left(\begin{array}{c} |X|\;-\;1\\ \left|S\right| \end{array}\right)}^{-1}\;\Delta_{PD}(S,\;i).$$

This index has certain appealing properties. In particular, $\sum_{i\in X}\psi_{i}^{\text{sh}}\;=\;PD(X)$, and there is a simple formula for quickly computing $\psi_{i}^{\text{sh}}$. The index *ψ*^sh^ also has a stochastic interpretation, but this is not based on extinction or survival of species, rather on the expected contribution to *PD* of each species under all possible orderings of the total set of species (for details see Haake et al. 2005). The index *ψ*^sh^ allocates existing *PD* ‘fairly’ amongst the species, whereas *ψ* quantifies the expected contribution of each species to future *PD*.

## Computing the HED Index

Computing the HED index directly via (1) could be problematic as it requires summation over all the subsets of *X* – {*i*} and this grows exponentially with |*X* |. However we now show that the index can be readily and quickly computed, both for rooted and unrooted trees. This polynomial-time algorithm for computing *ψ* thus complements (but is quite different to) the polynomial-time algorithm described by Haake et al. (2005) for computing *ψ*^sh^.

### Rooted trees

For a rooted phylogenetic *X*–tree T, and one of its edges *e*, let *C* (*e*) denote the set of species in *X* that are descended from *e* (i.e. the clade that results from deleting *e* from T). For *i* ∈ *X*, let *e*_1_, *e*_2_, ..., *e_k_* (*k* = *k*(*i*) ≥1) denote the edges (branches) on the path from *i* to the root of T, listed in the order they are visited by that path. Recall that *l*(*e*) denotes the length of edge *e*. The proof of the following theorem is given in the Appendix.

## Theorem 3.1

$$\psi_{i}\;=\;\sum_{r=1}^{k}l(e_{r})\prod_{j\in C(e_{r})-\{i\}}{\text{ɛ}}_{j}$$

Note that in this (and the next) theorem we adopt the convention Π *_j_*_∈Ø_ ɛ*_j_* = 1, which is relevant for the first term (*r =* 1) in the sum as *C* (*e_r_*) *–* {*i*} is empty. Thus the first term in the summation expression for *ψ_i_* given by Theorem 3.1 is simply *l*(*e*_1_)*,* the length of the pendant edge of T incident with species *i.*

### Example

We can apply the HED index to the members of the rooted tree depicted in Figure 1. For example, to compute *ψ_A_* by using Theorem 3.1 we have *ψ_A_* = 1 + 1 · **ɛ***_B_* + 1 · **ɛ***_B_***ɛ***_C_* = 1.19. By inspection, we can see that the most valuable species will be *D*, since it shares an edge with only one other species above the root, and that this species (*E*) has a high *P*(*ext*). At the other extreme, *A* shares its path to the root with two other species, and one of them (*B*) has a low *P*(*ext*). It should therefore receive a low HED value. The computed values are *ψ_D_* = 2.9, *ψ_B_* = 2.71, *ψ_E_* = 2.1, *ψ_C_* = 2.09, and *ψ_A_* = 1.19. Using the Shapley index (Haake et al. 2005), *D* and *E* are ranked first (with value = 2.63), followed by *C* (2.33) and then *A* and *B* (1.75). Pavoine’s QE metric (Pavoine et al. 2005) returns the same ranking as does the Shapley. A portal for computing HED is available at http://www.disconti.nu/-phylo/emd.dpf

### Unrooted trees, and properties of the index

We now provide a similar formula for efficiently computing the HED index for unrooted trees. Given a leaf *i* of T and an edge *e* of T, *e* induces a split of *X* into two disjoint subsets, and one of these subsets, which we denote as *C_i_* (*e*), contains *i*. The proof of the following theorem is given in the Appendix.

## Theorem 3.2

$$\psi_{i}\;=\;\sum_{e\in E(T)}l(e)\;\cdot \;\left(\prod_{j\in C_{i}(e)-\{i\}}{\text{ɛ}}_{j}\right)\;\cdot \;\left(1\;-\;\prod_{j\in X-C_{i}(e)}{\text{ɛ}}_{j}\right)$$

Notice that the rooted HED index is just a special case of the unrooted HED index (indeed Theorem 3.1 can be deduced from Theorem 3.2). To see this, given a rooted tree T attach a new leaf *ρ* to the root via a new edge to obtain an unrooted tree, and assign the new edge length *0.* Let **ɛ***_ρ_* *= 0.* Then it is easily seen that the HED index for T is just the HED index for the derived unrooted tree.

Using Theorem 3.1 it can be shown that if T is a rooted phylogenetic tree then the condition:

(3) $$\sum_{i\in X}\psi_{i}\;=\;PD(X),$$

holds for all selections of positive branch lengths and **ɛ**’s if and only if T is a ‘star tree’ (that is, every leaf is adjacent to the root). Moreover Theorem 3.2 shows that there is no unrooted phylogenetic tree T for which (3) holds for all positive branch lengths and ɛ values (of course (3) may hold on phylogenetic trees—either rooted or unrooted—if the branch lengths and **ɛ** values take certain values). This contrasts with the index *ψ*^sh^ which satisfies $\sum_{i\in X}\psi_{i}^{\text{sh}}\;=\;PD(X)$ on all unrooted phylogenetic trees and choices of branch lengths, a property that is referred to as the Pareto efficiency axiom by Haake et al. (2005). In the setting of this paper we should not be surprised that (3) holds for *ψ* only in very special cases since we are not trying to divide out existing *PD* amongst present taxa (one motivation behind *ψ*^sh^) but rather quantify the expected contribution each species makes to future *PD*.

## Application

**Figure 2.**
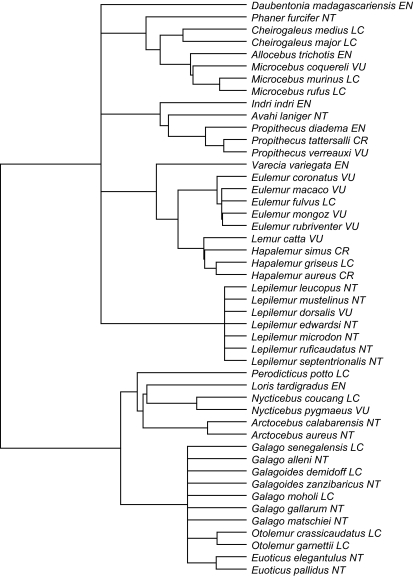
Prosimian species tree and associated IUCN threat categories. *CR*: critically endangered, *P* (*ext*) = 0.9; *EN*: endangered, *P* (*ext*) = 0.5; *VU*: vulnerable, *P* (*ext*) = 0.1; *NT*: near threatened, *P* (*ext*) = 0.01; *LC*: least concern, *P* (*ext*) = 0.001. Edge lengths are on an arbitrary scale that represents time since divergence.

We compared the HED index with the Shapley (Haake et al. 2005) values for the Prosimians (Mammalia: Primata), a group of approximately 50 species with a broad range of extinction probabilities. This group includes the Aye-Aye, the lemurs, the lorises and galagos. We made use of a recent dated Supertree of the order Primates (Vos and Mooers, 2004; Vos, 2006), see Figure 2, and Red List risk designations from the IUCN (www.iucnredlist.org, accessed February 2006). Following Isaac et al. (2007) and Redding and Mooers (2006), we first converted the five categories of risk (CR, EN, VU, NT, and LC) to probabilities of extinction. Under the IUCN criteria, the species in the VU category are given a *P*(*ext*) = 0.1 over the next 100 years. We gave the lowest and highest threat categories very conservative probabilities of extinction over the next 100 years of 0.001 and 0.9 respectively, leaving *P*(*ext*) = 0.5 for EN, and *P*(*ext*) = 0.01 for NT: this scale is very similar to that calculated from real population viability analyses for birds (Redding and Mooers, 2006). We are primarily interested in how the ranking of species changes using different approaches.

The bivariate correlation between the metrics is high (0.94). Both measures chose the Aye-Aye (*Daubentonia madagascarensis*) as the most important species, followed by *Perodicticus potto*. Interestingly, the three most highly ranked species under current conservation policy (the critically endangered lemurs *Propithecus tattersalli, Hapalemur simus, H. aureus*) are nested well up in the tree (Fig. 2) such that none of them were chosen in the top ten for either SV or HED. If we compare the rest of the rankings for these two metrics, the largest single difference is for the two *Arctocebus* species: they rank twelfth under SV (being relatively isolated on the tree), but only twenty-sixth under HED: because neither is severely threatened, the chances are good that their common path will persist.

On the prosimian tree, both measures are heavily influenced by the pendant edge (PE) length of the focal species (with correlations of PE vs. SV = 0.94, and of PE vs. HED = 0.98). PE is always part of the marginal increase to *ψ*, while interior edges are most likely represented with high probability, especially for larger and more balanced trees. Here, both polytomies and the use of a pure birth model for estimating unknown edge lengths bias pendant edges to be long. Simulated trees under more realistic models return significant but weaker correlations (e.g. for *d* = 0.9*b*, *N* = 500 species and a right-skewed distribution of extinction probabilities, correlations of PE vs. SV ~ 0.82, and PE vs. HED ~ 0.84). Even here, PE is a poor predictor of HED for the genus *Propithecus*, for *H. griseus*, and for *Nycticebus couang* (Fig. 2). The first two groups contain the three most endangered species, increasing the value of close relatives. *Nycticebus* is an isolated genus, and *N. couang*’s sister species is listed as vulnerable (*P*(*ext*) = 0.1). Likewise for *P. verreauxi* —although it has close relatives and so a short PE, these relatives are at high risk of extinction, which increases its value; this is what we saw with species *D* in Figure 1.

### Incorporating the focal taxon’s extinction risk (HEDGE scores)

The effect of close relatives’ risk status on one’s own value is precisely the strength of the HED approach. However, the fact that the extinction risk of other species affects a focal species, but its own risk does not is somewhat counterintuitive. We address this by showing how it is possible to write the HED index as the sum of two terms each of which takes into account the extinction risk of the focal species. To describe this further, let *I* be the random variable which takes the value *i* if the focal species *i* survives (at the future time under consideration) and which otherwise takes the value of the emptyset (i.e. ∅) if *i* goes extinct.

Let

$${\psi^{\prime }}_{i}\;=\;E\;\left[PD\left({\text{S}}_{i}\;\cup \;\{i\}\right)\;-\;PD\left({\text{S}}_{i}\;\cup \;I\right)\right],$$

where, as before ${\text{S}}_{i}$ is the random subset of species in *X* – {*i*} that survive. In words, *ψ*′*_i_* is the increase in the expected PD score if we condition on the event that species *i* survives. This quantity has also been investigated recently by Dan Faith (Faith, 2007) where it is referred to as (*expected*) *PD complementarity*.

Similarly, let

$${\psi^{\prime \prime }}_{i}\;=\;E\left[PD({\text{S}}_{i}\;\cup \;I)\;-\;PD\left({\text{S}}_{i}\right)\right].$$

In words, *ψ*″*_i_* is the decrease in the expected PD score if we condition on the event that species *i* becomes extinct. The following result describes how to compute these two indices easily from the HED index, and verifies that they add together to give the HED index (its proof is given in the Appendix).

## Theorem 4.1

- *ψ****′****_i_* *=* **ɛ***_i_* *· ψ_i_*,
- *ψ****″****_i_* *=* (1 *–* **ɛ***_i_*) *· ψ_i_,*
- *ψ****′****i + ψ****″****_i_* *= ψ_i_*.

The approach of assigning a value to a species which is a function of its phylogenetic distinctiveness and its extinction probability has been referred to as ‘expected loss’ by Redding and Mooers (2006) and, more evocatively, an ‘EDGE’ score (Evolutionarily Distinct and Globally Endangered) by Isaac et al. (2007).

In the same spirit we will call *ψ*′ and *ψ″* (which extend our HED index *ψ*) HEDGE (*heightened evolutionary distinctiveness and globally endangered*) scores. The HEDGE score *ψ*′ *_i_* is more relevant when evaluating actions that might save species, whereas the HEDGE score *ψ*″*_i_* is appropriate when evaluating actions that might cause the extinction of species (such as building a dam). Our measures link species-specific EDGE-type scores (Isaac et al. 2007) with the complementarity framework strongly advocated by Faith (2007).

One potential advantage of HED and HEDGE over previous scores is their flexibility in designing conservation scenarios. So for instance, we can choose IUCN-ranked species for which conservation is cheap and/or already partially successful, set their *P*(*ext*) to 0, and see how rankings of other species change. Alternatively, we might want to increase the *P*(*ext*) to 1.0 for certain species to see how others are affected.

Most generally, HED and HEDGE could be incorporated in an assessment of species value that included many factors besides risk and future contribution, e.g. the ecological, distributional and aesthetic values enumerated by Avise (2005), and the costs of recovery and probability of its success. We present these metrics in the hope that they will be used to promote the preservation of species and their natural habitats.

## Appendix: Proofs of theorems

### Proof of Theorem 3.1

First observe that the only edge lengths that contribute to Δ*_PD_* (*S*, *i*) are those from the set {*l*(*e*_1_), *l*(*e*_2_), …, *l*(*e**_k_*)}. Consequently, for the random set ${\text{S}}_{i}$ of surviving species of *X* – {*i*} we have

$$\Delta_{PD}({\text{S}}_{i},\;i)\;=\;\sum_{r\geq 1}l(e_{r})\cdot \;I_{r}({\text{S}}_{i})$$

where *I**_r_* ( ${\text{S}}_{i}$) is the 0,1 indicator random variable that takes the value 1 precisely if *e**_r_* is not an edge of the subtree of T connecting the taxa in ${\text{S}}_{i}$ and the root of T; since this is the only situation for which *e**_r_* lies in the subtree of T connecting ${\text{S}}_{i}$ ∪ {*i*} but not in the subtree of T connecting ${\text{S}}_{i}$. Thus, by linearity of expectation,

$$\begin{array}{l} \psi_{i}\;=\;E[\Delta_{PD}({\text{S}}_{i},\;i)]\;=\;E\left[\sum_{r\geq 1}l(e_{r})\;\cdot \;I_{r}({\text{S}}_{i})\right]\\ \;\;\;\;\;\;\;=\;\sum_{r\geq 1}l(e_{r})\;\cdot \;E[I_{r}({\text{S}}_{i})], \end{array}$$

and since *I**_r_* ( ${\text{S}}_{i}$) takes the values 0 and 1, E [*I**_r_* ( ${\text{S}}_{i}$)] = P [*I**_r_* ( ${\text{S}}_{i}$) = 1].

Thus,

$$\psi_{i}\;=\;\sum_{r\geq 1}l(e_{r})\;\cdot \;P[I_{r}({\text{S}}_{i})\;=\;1].$$

Now, the event ‘*I**_r_*( ${\text{S}}_{i}$) = 1’ occurs precisely if none of the elements in *C* (*e**_r_*) − {*i*} survive, and this latter event has probability Π*_j_*_∈_*_C_* _(_*_e_r__*_)− {_*_i_*_}_ ɛ*_j_*. Substituting this into the previous equation establishes the theorem.

### Proof of Theorem 3.2

For *i ∈ X* and the random sub ${\text{S}}_{i}$ ⊆ *X* –{*i*}, we have

$$\Delta_{PD}(S_{i},\;i)\;=\;\sum_{e\in E(\text{T})}l(e)\;\cdot \;I_{e}({\text{S}}_{i})$$

where *I**_e_*( ${\text{S}}_{i}$) is the 0,1 indicator random variable taking the value 1 precisely if ${\text{S}}_{i}$ consists of no elements of *C**_i_* (*e*) – {*i*} and at least one element of *X – C**_i_*(*e*).

Thus,

$$\psi_{i}\;=\;\sum_{e\in E(\text{T})}l(e)\;\cdot \;P[I_{e}({\text{S}}_{i})\;=\;1]$$

and by the independence assumption

$$\begin{array}{l} P[I_{e}({\text{S}}_{i})\;=\;1]\;=\;P\;[{\text{S}}_{i}\;\cap \;(C_{i}(e)\;-\;\{i\})\;=\;\emptyset ]\\ \;\;\;\;\;\;\;\;\;\;\;\;\;\;\;\;\;\;\;\;\;\;\;\;\;\;\;\;\;\;\;\;\;\;\;\;\times \;P\;[{\text{S}}_{i}\;\cap \;(X\;-\;C_{i}(e))\;\neq \;\emptyset ]. \end{array}$$

and so

$$P[I_{e}({\text{S}}_{i})\;=\;1]\;=\;\left(\prod_{j\in C_{i}(e)-\{i\}}{ɛ}_{j}\right)\;\cdot \;\left(1\;-\;\prod_{j\in X-C_{i}(e)}{ɛ}_{j}\right),$$

as claimed.

### Proof of Theorem 4.1

By definition,

$$\psi_{i}\;=\;E[PD({\text{S}}_{i}\;\cup \;\{i\})\;-\;PD({\text{S}}_{i})],$$

and so

$$E[PD({\text{S}}_{i}\;\cup \;\{i\})]\;=\;\psi_{i}\;+\;E[PD({\text{S}}_{i})].$$

Now we can write the unconditional expectation E [*PD*( ${\text{S}}_{i}$ ∪ *I* )] as the weighted sum of conditional expectations E [*PD*( ${\text{S}}_{i}$ ∪ *I* ) | *I* = {*i*}] P (*I* = {*i*}) + E [*PD*( ${\text{S}}_{i}$ ∪ *I* ) | *I* = ∅ ] P (*I* = ∅)

and so

$$\begin{array}{l} E[PD({\text{S}}_{i}\;\cup \;I)]\;=\;(1\;-\;{ɛ}_{i})\;E[PD({\text{S}}_{i}\;\cup \;\{i\})]\\ \;\;\;\;\;\;\;\;\;\;\;\;\;\;\;\;\;\;\;\;\;\;\;\;\;\;\;\;\;\;\;\;\;\;\;\;\;\;\;\;\;+\;{ɛ}_{i}E[PD({\text{S}}_{i})]. \end{array}$$

Parts (i) and (ii) now follow by applying these equations (and the linearity of expectation) to the definitions of *ψ*′*_i_* and *ψ*″*_i_* . Part (iii) follows directly from parts (i) and (ii).
